# Supplementary figures and images for: Network Approaches to Integrate Analyses of Genetics and Metabolomics Data with Applications to Fetal Programming Studies
Source: Metabolites. 2022 Jun 2;12(6):512. doi: 10.3390/metabo12060512 (PMC9229972; doi:10.3390/metabo12060512)

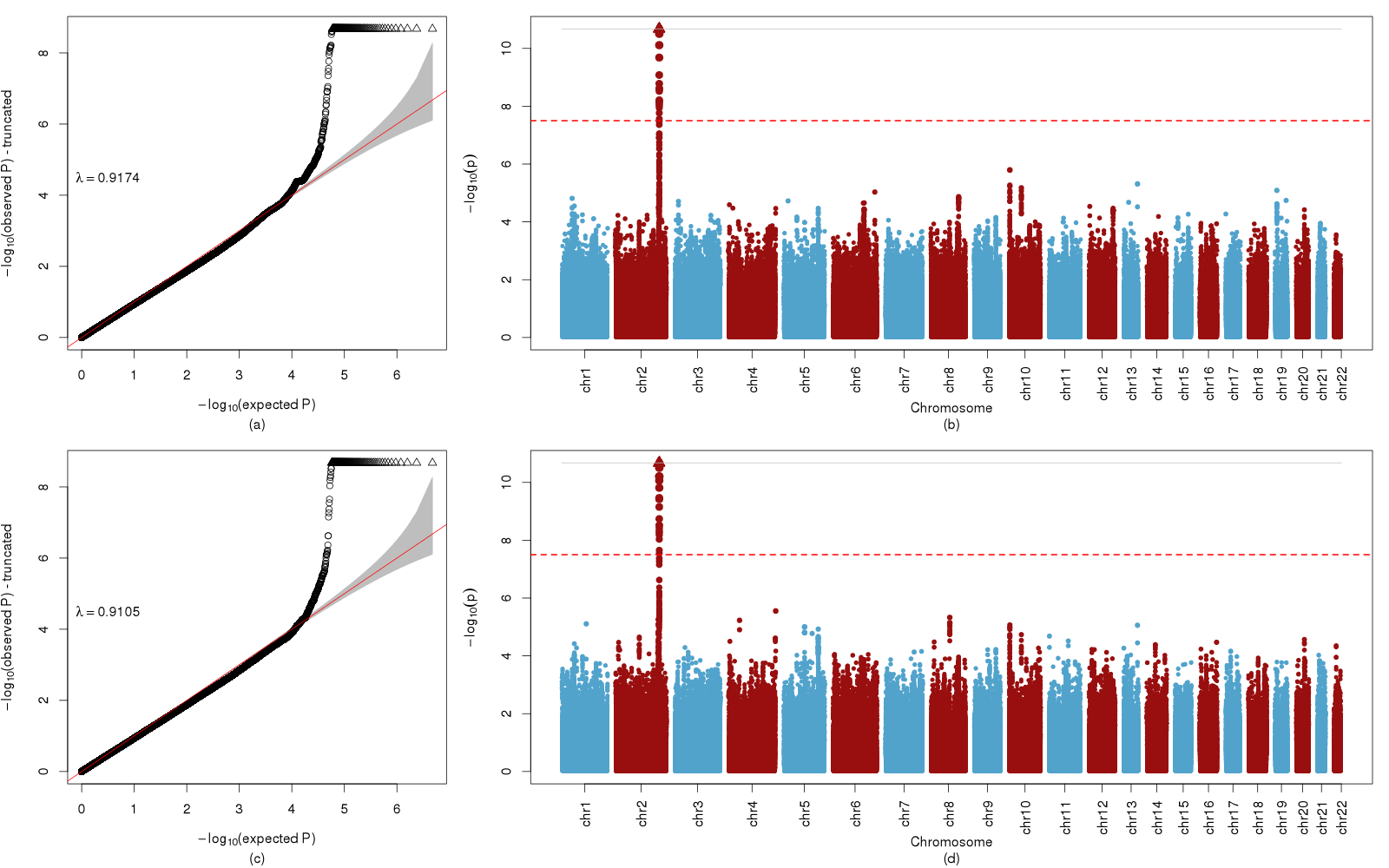

Supplement: Supplementary file 1 [file metabolites-12-00512-s001.zip › Supplement Figure S1 gly_aa.tiff]

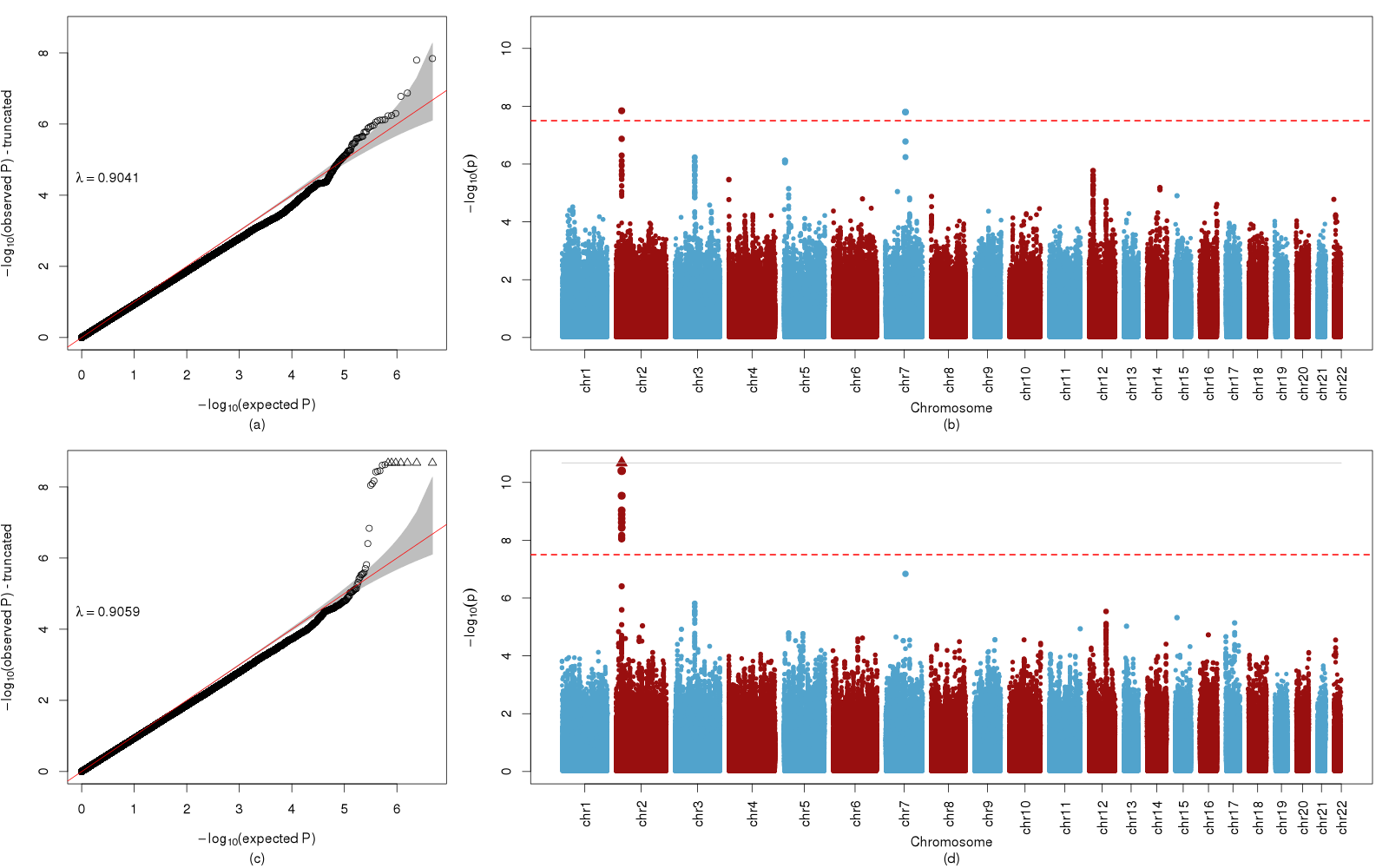

Supplement: Supplementary file 1 [file metabolites-12-00512-s001.zip › Supplement Figure S2 CAS_565_70_8_nt.tiff]

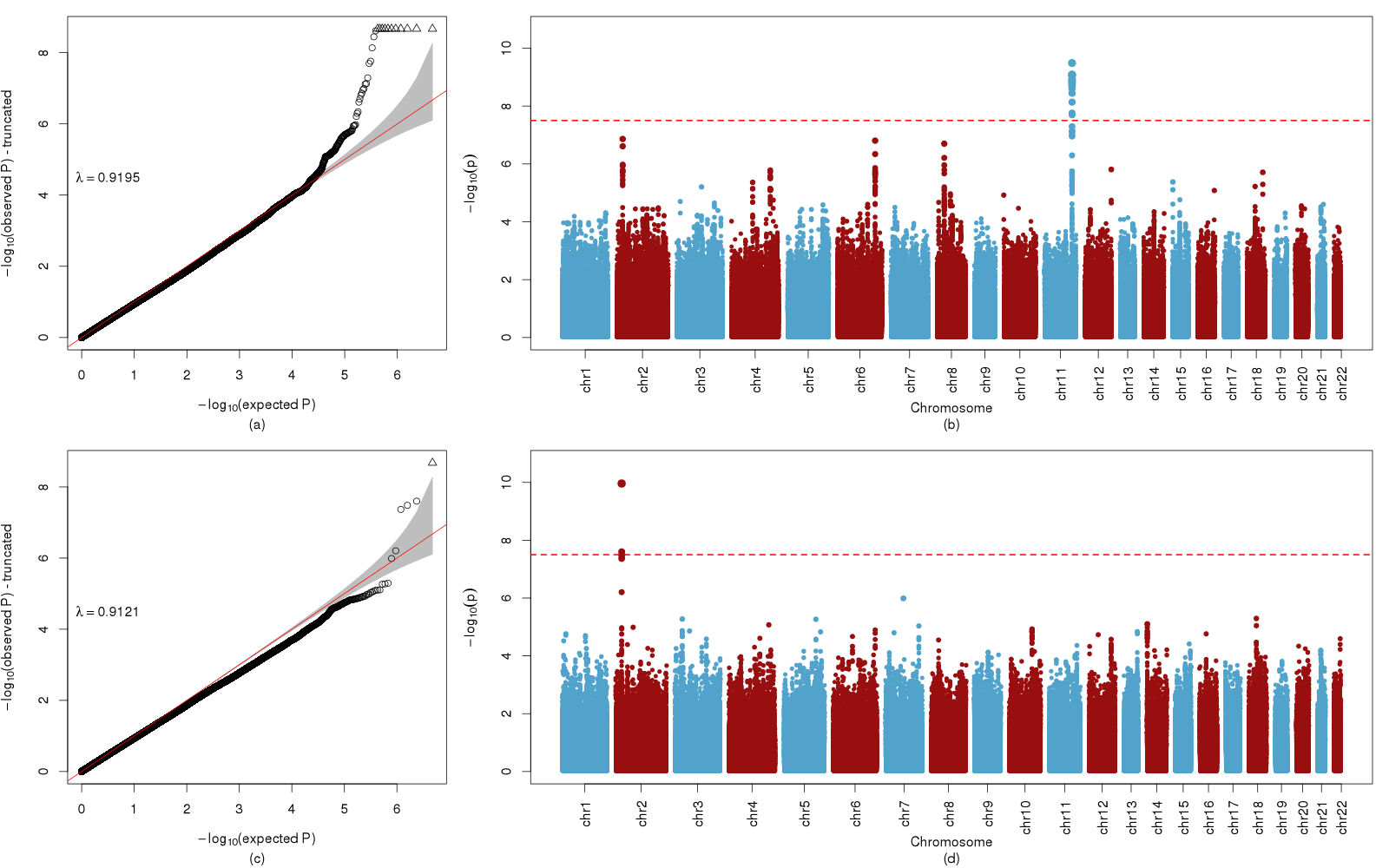

Supplement: Supplementary file 1 [file metabolites-12-00512-s001.zip › Supplement Figure S3 triglycerides_01_lactate_12.tiff]

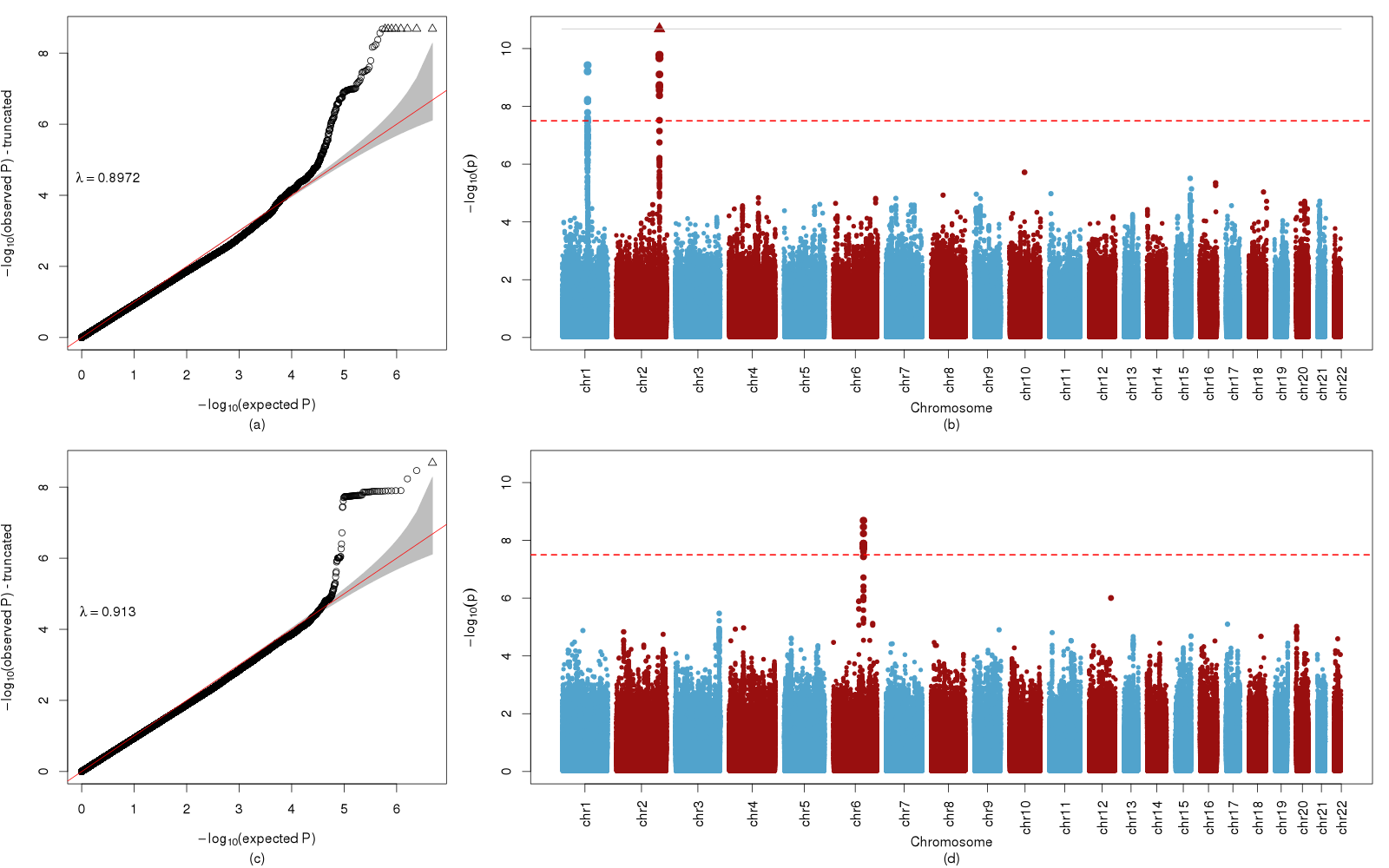

Supplement: Supplementary file 1 [file metabolites-12-00512-s001.zip › Supplement Figure S4 log_ac08_nefa_41.tiff]
